# PM_2.5_ Pollutant in Asia—A Comparison of Metropolis Cities in Indonesia and Taiwan

**DOI:** 10.3390/ijerph16244924

**Published:** 2019-12-05

**Authors:** Widya Liadira Kusuma, Wu Chih-Da, Zeng Yu-Ting, Handayani Hepi Hapsari, Jaelani Lalu Muhamad

**Affiliations:** 1 Department of Geomatics Engineering, Institut Teknologi Sepuluh Nopember (ITS), Surabaya 60111, Indonesia; liadira13@mhs.geodesy.its.ac.id (W.L.K.); hapsari@geodesy.its.ac.id (H.H.H.); 2 Department of Geomatics, National Cheng Kung University, Tainan 70101, Taiwan; chidawu@mail.ncku.edu.tw; 3 National Health Research Inst., No. 35, Keyan Rd, Zhunan, Miaoli County 35053, Taiwan

**Keywords:** air pollutions, fine particulate matter (PM_2.5_), GIS, remote sensing, land use regression (LUR)

## Abstract

Air pollution has emerged as a significant health, environmental, economic, and social problem all over the world. In this study, geospatial technologies coupled with a LUR (Land Use Regression) approach were applied to assess the spatial-temporal distribution of fine particulate (PM_2.5_). In-situ observations of air pollutants from ground monitoring stations from 2016–2018 were used as dependent variables, while the land-use/land cover, a NDVI (Normalized Difference Vegetation Index) from a MODIS sensors, and meteorology data allocations surrounding the monitoring stations from 0.25–5 km buffer ranges were collected as spatial predictors from GIS and remote sensing databases. A linear regression method was developed for the LUR model and 10-fold cross-validation was used to assess the model robustness. The *R*^2^ model obtained was 56% for DKI Jakarta, Indonesia, and 83% for Taipei Metropolis, Taiwan. According to the results of the PM_2.5_ model, the essential predictors for DKI Jakarta were influenced by temperature, NDVI, humidity, and residential area, while those for the Taipei Metropolis region were influenced by PM_10_, NO_2_, SO_2_, UV, rainfall, spring, main road, railroad, airport, proximity to airports, mining areas, and NDVI. The validation of the results of the estimated PM_2.5_ distribution use 10-cross validation with indicated *R*^2^ values of 0.62 for DKI Jakarta and 0.84 for Taipei Metropolis. The results of cross-validation show the strength of the model.

## 1. Introduction

Ambient air pollution has been related to increased levels of mortality and morbidity in megacities [1]. It is closely related to people’s daily lives. The relatively high growth of socio-economic, industrial, and urbanization activities in urban and suburban areas has great potential for increasing energy consumption, which is one of the factors of air pollution. There are several types of outdoor air pollution, such as Cox, NOx, Sox, Ox, and one of the pollutants that is quite dangerous for human health is PM_2.5_. PM_2.5,_ usually called fine particulate matter, is an air pollutant consisting of a mixture of solid and liquid particles. PM_2.5_ has a diameter of less than 2.5 μm, and thus, it is often referred to as smooth PM, which also consists of ultrafine particles with a diameter of less than 0.1 μm [2]. Epidemiological research associated with the study of PM_2.5_ exposure to the incidence of lung cancer, respiratory problems, and early death has shown a positive association [3,4]. The results show that it harms human health.

Several studies have shown that the concentrations of PM (particulate matter) can be affected by geographical features such as land use information, meteorology, and satellite data, including temporal variation parameters [5]. Methods for evaluating local urban variability to fine particulate matter were needed for these studies. Various models have been established around the world to explore the statistical correlations between ground monitoring stations of PM_2.5_ and the variables derived from geographical data information due to develops in the performance of GIS (Geographical Information System) technology.

Research is being conducted on the impact of geographical parameters on improvement air quality has been carried out. However, it is a big challenge to access PM2.5 data, particularly in developing countries. During the last period, some methods have been established to tackle challenges related to air pollution, such as interpolation using kriging and IDW (Inverse Distance Weighting) [6,7], and LUR (Land Use Regression) [8,9,10]. The interpolation of pollutant concentrations is based on the monitoring sites with densely clustered stations, whereas it is difficult to monitor locations with few stations. LUR models have proven to be relevant for these approaches in recent years. The method is to develop statistical regression models based on GIS platforms. These can be used to estimate air pollutant levels in a particular site by establishing a statistical correlation between pollutant observations and potential prediction variables [11,12,13].

In major cities, the types of land use can affect the level of PM through urban area development [14]. The land changes of forest, grasslands, agriculture in residential areas, industrial sites, and commercial centers frequently lead to increased emission levels. Air pollution concentration is related to changes in meteorological conditions. The development of prediction method starts by analyzing periods of serious atmospheric pollution, which are correlated with meteorological conditions monitored during those periods. These elements are considered as predictors [15]. Meteorological conditions such as wind speed, wind direction, and rainfall have an impact on particulate matter exposure [16]. Furthermore, satellite data using remote sensing, such as identification of greenness information, are efficient for measuring a wide range with multitemporal variations and easily available. The Normalized Difference Vegetation Index (NDVI) from Moderate Resolution Imaging Spectroradiometer (MODIS) sensors have been commonly used in greenness inventory management, which is a satellite-based method that has established a strong dynamic range and responsiveness for recording and calculating spatial-temporal variations in vegetation density [17,18]. NDVI has been used to determine green space area, which has a negative correlation with particulate matter [19].

Indonesia, with an area 1,922,570 km² (land only), has five ground stations monitoring PM_2.5_ concentration, one of the station locations is in DKI Jakarta [20]. In contrast, Taiwan, with an area of 36,000 km², has 78 ground stations to monitor air quality concentrations. For controlling and preventing air pollution from exceeding air quality standards, monitoring mass concentrations and multi-element identification of PM_2.5_ can be used for particle characterization and estimation of pollutant sources. In general, air quality monitoring, especially for PM_2.5_ is performed in various major cities in each country.

This study compares the air quality DKI Jakarta, in Indonesia and the Taipei Metropolis, in Taiwan using LUR model development for land use, meteorology, and greenness related to PM_2.5_. All the parameters have multi-temporal variability in order to achieve better performance in the estimation of PM_2.5_. The result acquired from this research will be particularly useful when developing LUR models in each country, in epidemiological studies and environmental health research in each country.

## 2. Materials and Methods

### 2.1. Study Area

The research area was in DKI Jakarta, in Indonesia and the Taipei Metropolis, in Taiwan, which are both capital cities. Taipei Metropolis includes 41 districts, with a total area of 2324 km^2^. The population densities of Taipei City and New Taipei City were 9818 and 1947 persons/km^2^, respectively [21]. According to the statistical data from the Land Use Investigation of Taiwan, Taipei Metropolis is covered by 68.43% forest, 8.44% buildings, 7.05% agricultural land, and 5.07% transportation infrastructure [22]. DKI Jakarta covers an area 662.33 km^2^, with 44 districts. However, only the major cities in DKI Jakarta were taken as the study area for Indonesia. The distribution of population density in DKI Jakarta was 15,763 persons/km^2^ for South Jakarta City, 15,385 persons/km^2^ for East Jakarta City, 19,143 persons/km^2^ for Central Jakarta City, 19,516 persons/km^2^ for West Jakarta City, and 12,146 persons/km^2^ for North Jakarta City [23]. Based on the classification from satellite data around 69.91% is built up area, 16.48% is mixed tree vegetation, 7.42% is grass land, and 1.63% is paddy field [24].

### 2.2. PM_2.5_ Concentration Data

**Figure 1:**
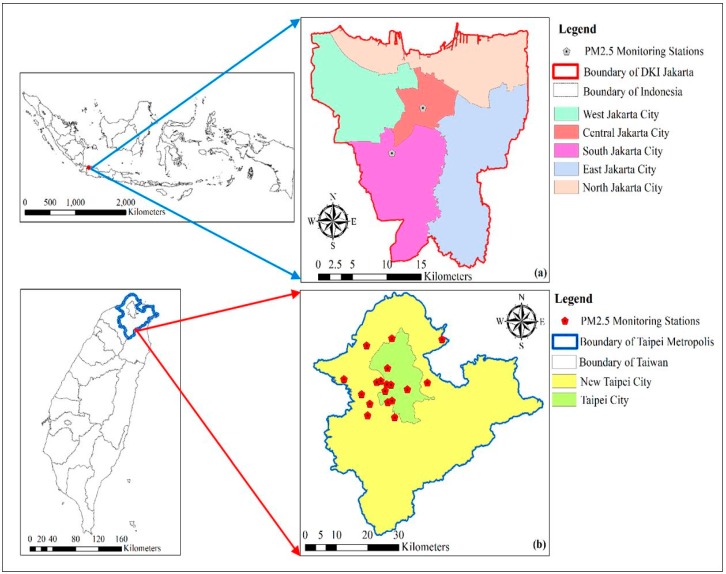
Distribution of PM_2.5_ monitoring stations, (**a**) in DKI Jakarta, Indonesia and (**b**) in Taipei Metropolis, Taiwan.

PM_2.5_ concentration data of PM_2.5_ in Jakarta, Indonesia, were obtained from the two monitoring stations through AirNow DOS, which collects PM_2.5_ concentration data from the U.S. Embassy in DKI Jakarta. In Taiwan, ground monitoring measurements of PM_2.5_ mass concentration in Taipei Metropolis were obtained from 17 automatic monitoring stations established by the Environmental Protection Administration Executive Yuan R.O.C (Taiwan) through the Taiwan Air Quality Monitoring Network. The stations distributed within the study area, including 14 general stations, two traffic stations, and one national park station, use beta ray analyzers. Daily concentration observations from 2016 to 2018 were aggregated into monthly averages. The illustrations of the study area and the monitoring stations are shown in Figure 1.

### 2.3. Geographic Information Datasets

**Figure 2:**
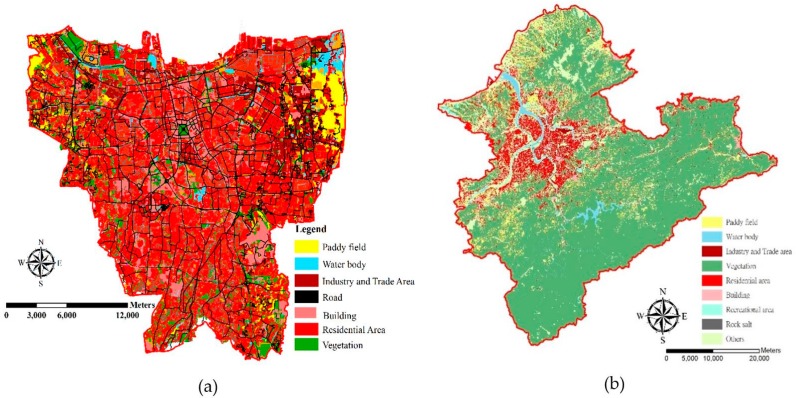
Land use map of (**a**) DKI Jakarta and (**b**) Taipei Metropolis.

Topography data for DKI Jakarta were provided by the Geospatial Information Agency. Daily meteorological data in DKI Jakarta were collected by Meteorological, Climatological, and Geophysical Agency (BMKG), which is a government agency. The data from monitoring stations on Java Island in Indonesia, including temperature, wind direction, wind speed, relative humidity, solar radiation, and rainfall, were obtained from the BMKG database center (Data Online—BMKG Database Center: http://dataonline.bmkg.go.id/home) from 1 January 2016 to 31 December 2018. Vegetation Indices (MOD13Q1) version 6 data are produced every 16 days at 250 × 250 m spatial resolution as a level 3 product by a Terra Moderate Resolution Imaging Spectroradiometer (MODIS). The algorithm chosen is based on the best pixel value from all acquisitions for each 16-day period, which include various criteria such as low clouds, low view angle, and the highest NDVI/EVI value [25]. Topography data for Taiwan can be accessed in open data provided by the government (https://data.gov.tw/en), the GIS-T Transportation Network Geographic Information Warehousing System (https://gist.motc.gov.tw/gist_web/GistMapGeneral/MapTopic), and the Geographic Information Map Cloud Service Platform (https://www.tgos.tw). Figure 2a describes the land use information in the big cities of DKI Jakarta, which is dominated by built up areas, such as residential areas, buildings, and industrial and trade areas. Figure 2b illustrates the land use information in Taipei Metropolis, which is dominated by vegetation, such as forest.

The estimated values of the predictor variables of the monitoring site coordinates were calculated through the GIS platform. The geospatial variables for land use and NDVI data were compiled in the range of a circular radius from 250 to 5000 m at each PM_2.5_ monitoring station to describe the area around it.

Data collection needed in this research included land use data in each region related to the land use data format with the shapefile format, meteorological data, and NDVI data from the MODIS sensor. NDVI data from the MODIS sensor can be downloaded at (https://ladsweb.modaps.eosdis.nasa.gov/search/order/1); the data downloaded only includes the area related to this study. The data processing step was then carried out after the data was collected, to create a database of land use data, meteorological data, and NDVI data. Land use data was formed by raster calculation of the focal statistics radius of 250–5000 m using ArcGIS and Python, for each type of land use. Meteorological data from each station point were obtained using the Inverse Distance Weighting method. All the raster maps (50 × 50 m^2^) were developed for the PM_2.5_ model to create each element based on the predictors, such as land use types, meteorology conditions, and NDVI. The focal statistics function in ArcMap was used to describe the predictor data for each type of land use and NDVI. Circular buffers with radiuses of 250–5000 m around each PM_2.5_ monitoring stations were used to generate maps.

### 2.4. LUR Modelling and Validation

LUR models were established for PM_2.5_ based on ground station monitoring data from the monitoring network and predictor variables. In addition, R x64 3.5.2 software (RStudio, Auckland, New Zealand) was used for statistical analysis, model development, and validating the models. ArcGIS 10.3 and Python were used to identify geographical variables (e.g., land use, meteorology, and NDVI) and create the map for prediction of PM_2.5_. The PM_2.5_ concentration models for DKI Jakarta and Taipei Metropolis were developed based on monthly variance from 1 January 2016 to 31 December 2018.

Stepwise linear regression is a combination of forward and backward selection techniques. This type of regression is used to calculate the percentage explanation variability in order to optimize the LUR models. Explanation of R^2^ was used for optimizing the percentage variability and assessing LUR models. The univariate regression analysis was carried out with the R^2^ performance and the coefficient correlations were listed for all predictor variables were listed in order to observe the direction and significance of the association. The LUR model with the highest R^2^ and the correct direction according to the predetermined criteria, was considered as the preliminary model. The high rank variable was selected and then the model was run to find the next significant variable. The Variance Inflation Factor (VIF) were examined to identify multicollinearity in the model development. When performing stepwise linear regression, the given conditions had values of ρ < 0.1 and VIF < 3 [19,26].

The PM_2.5_ prediction maps were generated for each cell with a resolution of 50 × 50 m using the regression Equation (1). The formula from the final result of the stepwise regression guidelines is shown as, follows:

(1) $$Y=\beta_{0}+\beta_{1}X_{1}+\beta_{2}X_{2}+\ldots +\beta_{n}X_{n},$$

where, Y is the PM_2.5_ concentrations; $\beta_{0}$ is the constant intercept of regression equation; $\beta_{1}$ to $\beta_{n}$ are regression coefficients; and $X_{1}$ to $X_{n}$ are the potential predictors of the central point.

Cross-validation was used to test the strength of the model. This study conducted 10-fold cross-validation. The 10-fold cross-validation was used to test the performance of the LUR model. The validation method determined by 90% of the data was used as training data, and the remaining 10% was used for validation [19]. The models were chosen with high R^2^, adj R^2^, and low RMSE for monthly concentrations.

## 3. Results

### 3.1. PM_2.5_ Concentrations between Indonesia and Taiwan

**Figure 3:**
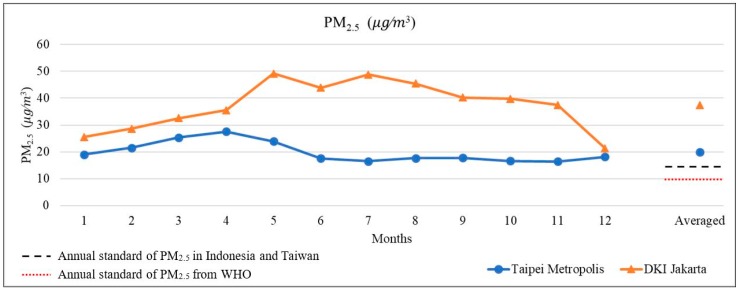
Comparison of time series trend of PM_2.5_ concentrations between DKI Jakarta and Taipei Metropolis from 2016 to 2018.

The results of the temporal trend are shown in Figure 3. According to the figure, the DKI Jakarta region has a higher level of pollution during the dry season than the rainy season, whereas for the Taipei City area has a high level of pollution in the spring season and a low level of pollution in the summer. Air quality guidelines (AQGs) with the base target level of PM_2.5_ is specified by the World Health Organization. The AQGs of PM_2.5_ for short-term (24-h average) and long-term (annual average) exposure are 25$\;\mu g∕m^{3}$ and 10 $\mu g/m^{3}$. Based on identified health effects, PM_2.5_ pollution index standard is used for long-term exposure. These are the lowest levels with a total increase in cardiopulmonary and lung cancer mortality of 95% to PM_2.5_ [27]. The guidelines on this matter have been established in Indonesia. The threshold of PM_2.5_ is 65 $\mu g/{\mathrm{Nm}}^{3}$ for the 24-h average and 15 $\mu g/{\mathrm{Nm}}^{3}$ for the annual average [28].

### 3.2. LUR Development

This study proposes two models of PM_2.5_ estimation, and the resulting model includes spatial-temporal PM_2.5_ estimation for DKI Jakarta, Indonesia, and Taipei Metropolis, Taiwan. The results of Spearman correlation were selected to look for variables that had high potential/correlation and had the same direction of correlation, for example, having a negative or positive correlation.

#### 3.2.1. PM_2.5_ Model in Indonesia

According to the results of correlation analysis, all variables selected have an intuitive relationship with PM_2.5_, such as variables related to residential areas that have a positive correlation and have a negative correlation with NDVI/Greenness. This study used a total of 496 variables. The criteria are shown in Table 1, along with the estimated efficiency and strength of the developed model. The indicated *R*^2^ value is 56% for variations of PM_2.5_ in DKI Jakarta, Indonesia. The results show that six variables were statistically significant predictors. Five variables, including temperature, NDVI with a radius of 1500 m, NDVI with a radius of 1750 m, humidity, and residential areas have values of ρ < 0.01, and NDVI with a radius of 4750 m has a value of ρ < 0.05. VIF was applied to assess the collinearity of the predictors in the developed model. The proposed the LUR model has a VIF value of less than 3. It shows that there were no cases of multicollinearity between predictor variables.

Descriptive statistical results are shown in Table 2 for the DKI Jakarta. The results were used to find information from the selected variables considered in this study.

#### 3.2.2. PM_2.5_ model in Taiwan

Based on the result of Spearman correlation analysis, all selected variable have powerful correlations, such as PM_10_, NO_2_, SO_2_, fall and spring season, UV, rainfall, major roads with a radius 250 m, railways with radius of 4000 and 5000 m, airports with nearest distance and radius 2500 and 5000 m, quarrying sites with radius 5000 m, and NDVI with a radius 4000 m, have powerful correlation. The UV, rainfall, NDVI, fall season, and airport with nearest distance variables have a negative correlation to determine the quality of PM_2.5_. This study used a total of 69 variables. The criteria are shown in Table 3, along with the estimated efficiency and strength of the developed model. The indicated R^2^ value is 84% for PM_2.5_ variations in Taipei Metropolis. The results show 14 variables are statistically significant predictors, 13 variables including PM_10_, NO_2_, SO_2_, UV, spring, main road, railroad, airport, airport closest distance, mine area, NDVI had a value of ρ < 0.01, and the level of rainfall has a value of ρ = 0.07. The VIF value of the LUR model has a value of less than 3. This shows that there are no cases of multicollinearity between predictor variables.

Descriptive statistical results are shown in Table 4 for Taipei Metropolis. The results were used to find information from the selected variables, which was used to develop the LUR model.

### 3.3. Model Performance

**Figure 4:**
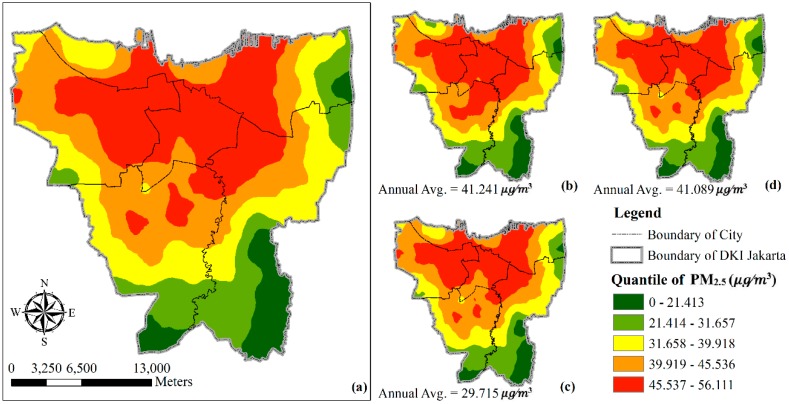
Prediction maps of spatial-temporal variability of PM_2.5_ concentration using the developed LUR model in DKI Jakarta: (**a**) 2016–2018, (**b**) 2016, (**c**) 2017, (**d**) 2018.

In this study, PM_2.5_ monthly estimation results of the DKI Jakarta area shown in Figure 4 have a high average PM_2.5_ value. PM_2.5_ in the region fluctuated, decreased in 2017, and showed high values in 2016 and 2018. The energy consumption of urban residents and the volume of vehicles continuously have an impact on urban air quality. The Normalized Difference Vegetation Index shows that it can reduce the level of PM_2.5_ in urban areas through a negative correlation.

**Figure 5:**
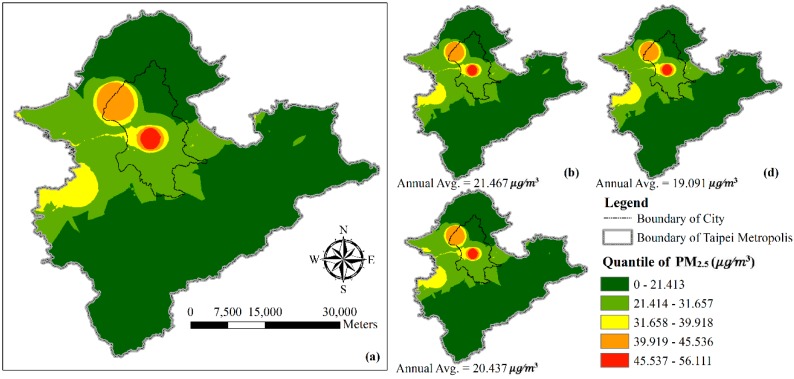
Prediction maps of spatial-temporal variability of PM_2.5_ concentration using the developed LUR model in Taipei Metropolis: (**a**) 2016–2018, (**b**) 2016, (**c**) 2017, (**d**) 2018.

Estimation of PM_2.5_ in the Taipei Metropolis area, shown in Figure 5, has a high average PM_2.5_ value. PM_2.5_ in the region has decreased from year to year, from 2016–2018. Urban population activity, airport emission, and the volume of vehicles continuously have an impact on urban air quality.

The range value (green to red) of PM_2.5_ was from 0–55.726 μg/m^3^. The value showed that density of PM_2.5_ particles was 55.726 μg/^3^, indicating the worst air quality. The resolution of the map shows the monthly averages from 2016–2018.

### 3.4. Model Validation

**Figure 6:**
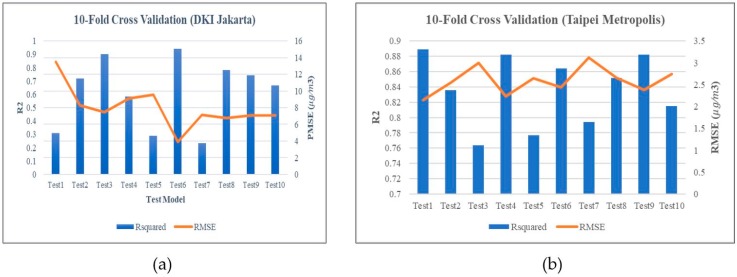
Result of 10-fold cross-validation of (**a**) DKI Jakarta and (**b**) Taipei Metropolis.

Figure 6 shows the comparison of R^2^, adj R^2^, and RMSE values. The results of R^2^ were 62% and 83% for DKI Jakarta and Taipei Metropolis, respectively. The 10-cross validation confirms the robustness of the PM_2.5_ model. Figure 6a shows that the average accuracy from 2016–2018 has an R^2^ value of 0.617 for DKI Jakarta. The validation shows that the model developed is entirely accurate. Figure 6b shows the comparison between model predictions and observations at each point in 2018. Figure 6 shows the comparison of predictions between DKI Jakarta and New Taipei City for PM_2.5_ in 2018.

## 4. Discussion

This study implemented land use, meteorology and greenness data and long-term PM_2.5_ monitoring data, from DKI Jakarta in Indonesia and Taipei Metropolis in Taiwan. The study covered the capital cities of the two countries. The variables were concerned with multi-temporal variability data. The LUR model has been suitable method for estimating the concentration of pollutants in several areas, especially for PM_2.5_ [9,11,12]. The model shows good predictive ability (R^2^ = 56% for DKI Jakarta, and 84% for Taipei Metropolis), with spatial resolution of 50 × 50 meters. LUR can be used to determine the relations between PM_2.5_ and various variables. The results were derived from the proposed two model development approaches. The model can be validated using 10-fold cross-validation which shows the model robustness. It can also be shown by the R^2^ value of cross validation (R^2^ = 56% for DKI Jakarta, and 84% for Taipei Metropolis). The results spatial-temporal maps can be obtained using the LUR model in each country.

A limitation of our study was the lack of traffic-related and satellite data (such as, AOD (Aerosol Optical Depth) from MODIS sensor) to determine the level of PM_2.5_ concentrations. Land use information in the two countries is based on different information standards, which could affect the number of variables used in selecting correlations. However, AOD (MCD19A2 products) from the MODIS sensor did not show pixel values, due to the cloud density/null value in the study area. It was difficult to obtain more specific GIS data for these sources.

However, this study showed that DKI Jakarta has a lower R^2^ value rather than Taiwan. This could be because to the total numbers of monitoring stations are not balanced between these regions. The small number of monitoring stations in DKI Jakarta could affect LUR model development due to the number of predictors factored, which might have influenced the performance of LUR model establishment. The varying distribution of station monitoring networks across countries could contribute to variations in PM_2.5_ level.

The proportion of residential areas, major roads, railways, airports, and quarrying sites has important effects across land use characteristics in the model of PM2.5. However, according to the land use data, residential areas showed a significant positive correlation with PM_2.5_ concentration in DKI Jakarta. Studies of PM, the correlation have demonstrated high correlation between PM_2.5_ level and residential areas, especially in high-density residential areas [29,30].

Several studies have demonstrated that meteorological factor (such as humidity, temperature, wind speed and wind direction) highly correlate with air pollution, espacially PM_2.5_ concentrations [31,32,33]. Statistical modelling of PM_2.5_ in previous studies consistently showed higher concentrations in spring periods compared to fall periods [34]. Coefficient estimation from the LUR model showed that PM_2.5_ has a positive correlation with spring periods and a negative correlation with fall periods.

NDVI was obtained from satellite data using MODIS sensors to calculate greenness in the research area. However, NDVI was not differentiated among greenness types (such as planted area, green spaces, public parks, etc.). The difference of greenness type might be relevant to air pollutants. Previous studies have shown that greenness can affect air pollution as well [35].The annual averages of PM_2.5_ in Taipei Metropolis and DKI Jakarta exceed the national policy threshold standard in Indonesia and Taiwan, which is 15 $\mu g∕m^{3}$. Meanwhile, the concentration of PM_2.5_ both countries also exceed the threshold standard of the WHO. Accurate and frequent ground monitoring stations of air quality concentrations are necessary for the capital cities in each country. They can be used to assess air quality, identify the most relevant potential sources, strengthen management to control air quality, and provide advice to policymakers, especially to the governments. By a adopting remote sensing and GIS techniques, a comprehensive strategy can be used to develop an air quality monitoring network, which allows for controlling air quality for the protection of human health [36].

## 5. Conclusions

This study analyzed recent trends of PM_2.5_ concentrations from 2016–2018 in two countries in Asia. LUR models were used to predict of PM_2.5_ level. The assessment of PM_2.5_ based on spatial-temporal was strongly influenced by land use, meteorological conditions, and MODIS NDVI. PM_2.5_ pollution in the Taipei Metropolis region, has a positive correlation with PM_10_, SO_2_, NO_2_, spring conditions, main roads, railways, airports, mining areas, and has a negative correlation with UV, rainfall, airports within close distances. PM_2.5_ pollution in DKI Jakarta, was strongly influenced by humidity, NDVI, temperature and residential areas. PM_2.5_ pollution in DKI Jakarta, has a positive correlation with residential areas, temperatures and has a negative correlation with NDVI, humidity. The R^2^ values of the resulting model were 0.84 and 0.56 for Taipei Metropolis and DKI Jakarta, respectively. Meanwhile, the 10-cross validation result shows R^2^ values of 0.83 and 0.61 for Taipei Metropolis and DKI Jakarta, respectively.

## Figures and Tables

**Table 1:** Coefficient estimates of the developed LUR (Land Use Regression) model for DKI Jakarta.

Variable	β	ρ	VIF	Partial R^2^	Model Performance
Intercept	−60.566	0.360			R^2^ = 0.56
Temperature	5.797	<0.001	1.57	0.325	ADJ R^2^ = 0.52
NDVI_1500 m_	−77.419	<0.001	1.07	0.098	RMSE = 8.19
NDVI_4750 m_	−54.467	<0.05	1.04	0.044	
NDVI_1750 m_	−50.729	<0.01	1.20	0.27	
Relative humidity	−0.779	<0.005	1.82	0.36	
Residential Area_4250 m_	0.039	<0.05	1.39	0.26	

**Table 2:** Descriptive statistics of the selected predictors.

Variable (Unit)	Min	Max	Standard Deviation	Variance	Medium (25–75% Percentile)
^a^ NDVI_1500 m_	1.43 × 10^−1^	3.36 × 10^−1^	5.27 × 10^−2^	2.79 × 10^−3^	0.256 (2.00 × 10^−1^–2.86 × 10^−1^)
^b^ NDVI_1750 m_	1.08 × 10^−1^	3.43 × 10^−1^	5.99 × 10^−2^	3.59 × 10^−3^	0.272 (2.29 × 10^−1^–2.93 × 10^−1^)
^c^ NDVI_4750 m_	1.35 × 10^−1^	2.97 × 10^−1^	4.22 × 10^−2^	1.79 × 10^−3^	0.236 (1.91 × 10^−1^–2.63 × 10^−1^)
Relative humidity	67.603	94.194	4.985	24.858	78.409 (75.741–80.981)
Temperature	30.357	33.872	7.43 × 10^−1^	0.5522	32.614 (32.085–32.897)
^d^ Residential area_4250 m_	337.099	451.852	57.778	3,338.41	394.476 (337.099–451.852)

^a^ Average NDVI within a radius of 1500 m; ^b^ Average NDVI within a radius of 1750 m; ^c^ Average NDVI within a radius of 4750 m; ^d^ Residential area within a radius of 4250 m.

**Table 3:** Coefficient estimates of the developed LUR model for Taipei Metropolis.

Variable	β	ρ	VIF	Partial R^2^	Model Performance
Intercept	1.978	0.16			R^2^ = 0.84
PM_10_	0.302	<0.001	2.37	0.56	ADJ R^2^ = 0.84
NO_2_	0.266	<0.001	2.98	0.09	RMSE = 2.57
SO_2_	2.101	<0.001	2.37	0.01	
UV	−0.326	<0.001	2.39	0.01	
Rainfall	−0.074	0.07	1.16	0.001	
Fall	−0.501	0.05	1.21	0.002	
Spring	1.828	<0.001	1.94	0.001	
Major Road_250 m_	6.42 × 10^−3^	<0.001	1.38	0.01	
Railway_4000 m_	1.151	<0.001	1.51	0.02	
Railway_5000 m_	0.615	<0.001	1.47	0.02	
Airport_2500 m_	0.092	<0.001	1.75	0.11	
Airport_5000 m_	0.043	<0.001	2.94	0.01	
Airport_nearest distance_	−1.03 × 10^−4^	<0.01	2.81	0.004	
Quarryingsite_5000 m_	0.346	<0.001	1.29	0.003	
NDVI_4000 m_	−4.46 × 10^−4^	<0.001	1.56	0.01	

**Table 4:** Descriptive statistics of the selected predictors of Taiwan.

Variable (Unit)	Min	Max	Standard Deviation	Variance	Medium (25–75% Percentile)
PM_10_	7.1	58.3	8.79	77.2	33.6 (30–41.9)
NO_2_	2.8	34.1	4.6	21.2	19.2 (17.1–22.1)
SO_2_	1.73	4.11	0.457	0.21	2.75 (2.45–3.13)
UV	1.83	10.62	2.35	5.51	5.9 (3.76–8.21)
Rainfall	0.04	14.39	2.68	7.2	2.28 (1.38–3.89)
Fall	0	1	0.434	0.189	0 (0–1)
Spring	0	1	0.432	0.187	0 (0–0)
^a^ Major Road_250 m_	0	586.4	144.8	20,989	30.9 (0–123.4)
^b^ Railway_4000 m_	0	2.863	0.709	0.502	0.124 (0–0.622)
^c^ Railway_5000 m_	0	6.29	1.97	3.9	0.318 (0–0.875)
^d^ Airport_2500 m_	0	77.8	17.9	320.5	0 (0–0)
^e^ Airport_5000 m_	0	54.7	19.7	387	0 (0–8.12)
^f^ Airport_nearest distance_	1433	17,289	4431.3	19,636,747	7356 (4425.5–11,242)
^g^ Quarryingsite_5000 m_	0	5.73	1.33	1.77	0 (0–0.318)
^h^ NDVI_4000 m_	585	9985	1335.3	1,783,200	8826 (7985–NA)

^a^ Major road within a radius of 250 m; ^b^ Railway within a radius of 4000 m; ^c^ Railway within a radius of 5000 m; ^d^ Airport within a radius of 2500 m; ^e^ Airport within a radius of 5000 m; ^f^ Airport within the nearest distance; ^g^ Quarry site within a radius of 5000 m; ^h^ Average NDVI within a radius of 4000 m.

## References

[1] Gurjar B.R., Jain A., Sharma A., Agarwal A., Gupta P., Nagpure A.S., Lelieveld J. (2010). Human health risks in megacities due to air pollution. Atmos. Environ..

[2] WHO (2013). Health Effects of Particulate Matter.

[3] West J.J., Cohen A., Dentener F., Brunekreef B., Zhu T., Armstrong B., Bell M.L., Brauer M., Carmichael G., Costa D.L. (2016). What We Breathe Impacts Our Health: Improving Understanding of the Link between Air Pollution and Health. Environ. Sci. Technol..

[4] Puett R.C., Hart J.E., Yanosky J.D., Spiegelman D., Wang M., Fisher J.A., Hong B., Laden F. (2014). Particulate Matter Air Pollution Exposure, Distance to Road, and Incident Lung Cancer in the Nurses’ Health Study Cohort. Environ. Health Perspect..

[5] Liu Y., Paciorek C.J., Koutrakis P. (2009). Estimating regional spatial and temporal variability of PM2.5 concentrations using satellite data, meteorology, and land use information. Environ. Health Perspect..

[6] Zhang G., Rui X., Fan Y. (2018). Critical review of methods to estimate PM 2.5 concentrations within specified research region. ISPRS Int. J. Geo-Inf..

[7] Li L., Gong J., Zhou J. (2014). Spatial interpolation of fine particulate matter concentrations using the shortest wind-field path distance. PLoS ONE.

[8] Beelen R., Hoek G., Pebesma E., Vienneau D., de Hoogh K., Briggs D.J. (2009). Mapping of background air pollution at a fine spatial scale across the European Union. Sci. Total Environ..

[9] Shairsingh K.K., Jeong C.H., Wang J.M., Brook J.R., Evans G.J. (2019). Urban land use regression models: Can temporal deconvolution of traffic pollution measurements extend the urban LUR to suburban areas?. Atmos. Environ..

[10] Xu S., Zou B., Lin Y., Zhao X., Li S., Hu C. (2019). Strategies of Method Selection for Fine Scale PM2.5 mapping in Intra-Urban Area Under Crowdsourcing Monitoring. Atmos. Meas. Tech..

[11] Lee J.H., Wu C.F., Hoek G., de Hoogh K., Beelen R., Brunekreef B., Chan C.C. (2015). LUR models for particulate matters in the Taipei metropolis with high densities of roads and strong activities of industry, commerce and construction. Sci. Total Environ..

[12] Hsu C.-Y., Wu C.-D., Hsiao Y.-P., Chen Y.-C., Chen M.-J., Lung S.-C.C. (2018). Developing Land-Use Regression Models to Estimate PM2.5-Bound Compound Concentrations. Remote Sens..

[13] Eeftens M., Beelen R., de Hoogh K., Bellander T., Cesaroni G., Cirach M., Declercq C., Dėdelė A., Dons E., de Nazelle A. (2012). Development of land use regression models for PM 2.5, PM 2.5 absorbance, PM 10 and PM coarse in 20 European study areas; Results of the ESCAPE project. Environ. Sci. Technol..

[14] Yang H., Chen W., Liang Z. (2017). Impact of land use on PM2.5 pollution in a representative city of middle China. Int. J. Environ. Res. Public Health.

[15] Berlyand M.E. (1991). Prediction and Regulation of Air Pollution.

[16] Kusumaningtyas S.D.A., Aldrian E., Wati T., Atmoko D., Sunaryo S. (2018). The recent state of ambient air quality in Jakarta. Aerosol Air Qual. Res..

[17] Huete A., Didan K., Miura T., Rodriguez E.P., Gao X., Ferreira L.G. (2002). Overview of the radiometric and biophysical performance of the MODIS vegetation indices. Remote Sens. Environ..

[18] Xiaoxia S., Zhengjun L. (2008). Vegetation Cover Annual Changes Based on Modis/Terra Ndvi in the Three Gorges Reservoir. Area. Remote Sens. Spat. Inf. Sci..

[19] Wu C.D., Chen Y.C., Pan W.C., Zeng Y.T., Chen M.J., Guo Y.L., Lung S.C. (2017). Land-use regression with long-term satellite-based greenness index and culture-specific sources to model PM2.5 spatial-temporal variability. Environ. Pollut..

[20] Kualitas Udara, Informasi Konsentrasi Partikulat (PM2.5). https://www.bmkg.go.id/kualitas-udara/informasi-partikulat-pm25.bmkg..

[21] MOI (Ministry of The Interior) (2019). Population for Township and District. https://www.moi.gov.tw/files/site_stuff/321/1/month/m1-07.xls.

[22] NLSC (Ministry of Land Surveying and Mapping) (2019). Statistics from 105 to 106 for Land Use Investigation of Taiwan. https://www.nlsc.gov.tw/LUI/Home/Content.aspx.

[23] Statistics of DKI Jakarta Province (2018). DKI Jakarta Province in Figures. https://jakarta.bps.go.id/publication/2018/08/16/67d90391b7996f51d1c625c4/provinsi-dki-jakarta-dalam-angka-2018.html.

[24] Rushayati S.B., Prasetyo L.B. (2016). Adaptation Strategy Toward Urban Heat Island at Tropical Urban Area. Procedia Environ. Sci..

[25] Didan K. (2015). MOD13Q1 MODIS/Terra Vegetation Indices 16-Day L3 Global 250m SIN Grid V006.

[26] Zhang Z., Wang J., Hart J.E., Laden F., Zhao C., Li T., Zheng P., Li D., Ye Z., Chen K. (2018). National scale spatiotemporal land-use regression model for PM2.5, PM10 and NO_2_ concentration in China. Atmos. Environ..

[27] World Health Organization (2006). WHO air quality guidelines for particulate matter, ozone, nitrogen dioxide and sulfur dioxide.

[28] Indonesian Government, Presiden republik indonesia,” Peratur Pemerintah Republik Indones. Nomor 41 Tahun 1999 Tentang Pengendali. Pencemaran Udar. http://jdih.pom.go.id/produk/PERATURAN%20PEMERINTAH/PP_No_28_th_2004%20plus%20penjelasan.pdf.

[29] Park S.H., Ko D.-W. (2018). Investigating the effects of the built environment on PM2.5 and PM10: A case study of Seoul Metropolitan city, South Korea. Sustainability.

[30] Han L., Zhou W., Li W. (2015). Increasing impact of urban fine particles (PM2.5) on areas surrounding Chinese cities. Sci. Rep..

[31] Tai A.P.K., Mickley L.J., Jacob D.J. (2010). Correlations between fine particulate matter (PM2.5) and meteorological variables in the United States: Implications for the sensitivity of PM2.5 to climate change. Atmos. Environ..

[32] Syafei A.D., Fujiwara A., Zhang J. (2014). Spatial and Temporal Factors of Air Quality in Surabaya City: An Analysis based on a Multilevel Model. Soc. Behav. Sci..

[33] Verma S.S., Desai B. (2016). Effect of Meteorological Conditions on Air Pollution of Surat City. J. Int. Environ. Appl. Sci..

[34] Chen W., Yan L., Zhao H. (2015). Seasonal Variations of Atmospheric Pollution and Air Quality in Beijing. Atmosphere.

[35] Givoni B. (1991). Impact of planted areas on urban environmental quality: A review. Atmos. Environ..

[36] Rotatori M., Salvatori R., Salzano R., Mazzeo N.A. (2011). Planning Air Pollution Monitoring Networks in Industrial Areas by Means of Remote Sensed Images and GIS Techniques. Air Quality Monitoring, Assessment and Management.

